# A Convenient Model of Severe, High Incidence Autoimmune Gastritis Caused by Polyclonal Effector T Cells and without Perturbation of Regulatory T Cells

**DOI:** 10.1371/journal.pone.0027153

**Published:** 2011-11-09

**Authors:** Eric Tu, Desmond K. Y. Ang, Thea V. Hogan, Simon Read, Cheryl P. Z. Chia, Paul A. Gleeson, Ian R. van Driel

**Affiliations:** Department of Biochemistry and Molecular Biology, Bio21 Molecular Science and Biotechnology Institute, The University of Melbourne, Parkville, Victoria, Australia; McGill University Health Center, Canada

## Abstract

Autoimmune gastritis results from the breakdown of T cell tolerance to the gastric H^+^/K^+^ ATPase. The gastric H^+^/K^+^ ATPase is responsible for the acidification of gastric juice and consists of an α subunit (H/Kα) and a β subunit (H/Kβ). Here we show that CD4^+^ T cells from H/Kα-deficient mice (H/Kα^−/−^) are highly pathogenic and autoimmune gastritis can be induced in sublethally irradiated wildtype mice by adoptive transfer of unfractionated CD4^+^ T cells from H/Kα^−/−^ mice. All recipient mice consistently developed the most severe form of autoimmune gastritis 8 weeks after the transfer, featuring hypertrophy of the gastric mucosa, complete depletion of the parietal and zymogenic cells, and presence of autoantibodies to H^+^/K^+^ ATPase in the serum. Furthermore, we demonstrated that the disease significantly affected stomach weight and stomach pH of recipient mice. Depletion of parietal cells in this disease model required the presence of both H/Kα and H/Kβ since transfer of H/Kα^−/−^ CD4^+^ T cells did not result in depletion of parietal cells in H/Kα^−/−^ or H/Kβ^−/−^ recipient mice. The consistency of disease severity, the use of polyclonal T cells and a specific T cell response to the gastric autoantigen make this an ideal disease model for the study of many aspects of organ-specific autoimmunity including prevention and treatment of the disease.

## Introduction

Autoimmune gastritis is an excellent system to investigate the loss of T cell tolerance to self-tissues since it is a well-characterised autoimmune disease with a known target antigen and the mouse model of the disease has many similarities to the human equivalent. Pernicious anemia, the end stage of autoimmune gastritis, has an estimated prevalence of 1.9% in Western adult population over the age of 60 years [1], which represents the commonest cause of vitamin B_12_ deficiency in the elderly [2]. Autoimmune gastritis features infiltration of mononuclear cells in the submucosa which extends into the gastric mucosa, the depletion of gastric parietal and zymogenic cells and hypertrophy of the gastric mucosa [1].

The target antigen in autoimmune gastritis has been identified as the gastric H^+^/K^+^ ATPase expressed by gastric parietal cells [3]–[5]. The gastric H^+^/K^+^ ATPase is a membrane proton pump responsible for the acidification of gastric juice and it is a heterodimer that consists of a catalytic α subunit (H/Kα) and a glycoprotein β subunit (H/Kβ). Previous studies have demonstrated that both H/Kα and H/Kβ are targeted in autoimmune gastritis [4], [6]–[8]. It is well documented that, in both mice and man, CD4^+^ T cells that recognise the gastric H^+^/K^+^ ATPase initiate autoimmune gastritis while CD8^+^ T cells and B cells are ineffective in doing so [7]–[17]. Nevertheless, following initiation of the disease, autoantibodies to the H^+^/K^+^ ATPase are produced and are a very useful diagnostic tool of the disease although they are not pathogenic [3], [16], [18].

BALB/c mice have been shown to be the most susceptible to lymphopenia-induced autoimmune gastritis with an incidence of 30–90% [19], [20]. The disease can be induced by thymectomy of 3 day-old mice or adult thymectomy combined with a single dose of cyclophosphamide [19], [21]. In spite of this, it is difficult to evaluate therapeutic effects of treatments given to thymectomised mice since a spectrum of disease severity from mild to severe is always observed and incidence is variable, thus large group sizes are required to test statistical significance. Furthermore, the surgery involved and post-operative maintenance of animals is technically demanding. Autoimmune gastritis can also be induced in BALB/c mice by immunisation with purified gastric H^+^/K^+^ ATPase [5]. However, the disease is reversible after cessation of immunisation and has a low severity. Transfer of BALB/c T cells depleted of regulatory T (Treg) cells into athymic recipient mice causes severe autoimmune gastritis [15]. It is therefore impossible to dissect the relationship between autoreactive T cells and Treg cells during the disease development in this setting since Treg cells are absent.

High incidence of spontaneous autoimmune gastritis is observed in transgenic mice expressing a TCR specific for H/Kα [8]. However, these mice do not represent an ideal disease model for all experiments because the monoclonal nature of the pathogenic T cells does not recapitulate the polyclonal response that occurs in pathophysiological conditions.

From this perspective, we developed a disease model which relied on the transfer of polyclonal T cells from H/Kα-deficient (H/Kα^−/−^) mice into sublethally-irradiated wildtype mice. We demonstrated that mice that received unfractionated T cells from H/Kα^−/−^ mice all succumbed to the most severe autoimmune gastritis with pathological changes observed throughout the whole stomach. Furthermore, these pathological changes significantly affected the physiology of stomach. We demonstrated the usefulness of this model by showing that the development of autoimmune gastritis in this model was strictly dependent on the presence of both H^+^/K^+^ ATPase α and β subunits.

## Results

### Elevated T and B cell response in H/Kα^−/−^ mice following immunisation with H^+^/K^+^ ATPase

In order to have a system that is suitable for the study of different aspects of autoimmunity, we established a disease model in which the disease is caused by pathogenic polyclonal T cells and the disease severity is consistently severe. A T cell response to H/Kα is required for the onset of autoimmune gastritis and may be the dominant source of gastric autoreactive epitopes [7], [8], [22]. As T cell tolerance to H/Kα was unlikely to occur in H/Kα^−/−^ mice, we investigated if T cells that were specific to H^+^/K^+^ ATPase and capable of causing the gastritis were present in H/Kα^−/−^ mice.

**Figure 1:**
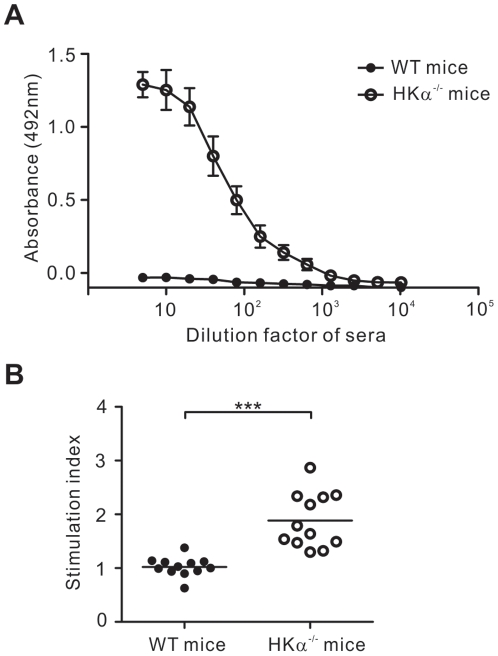
Elevated immune response to immunisation with H^+^/K^+^ ATPase in H/Kα^−/−^ mice. WT and H/Kα^−/−^ mice were immunised with H^+^/K^+^ ATPase as described in “Materials and Methods”. (A) H^+^/K^+^ ATPase-specific autoantibodies in serum were detected by ELISA using serial 2-fold dilutions of mouse sera, with a starting dilution factor of 5. Data is representative of three independent experiments. Error bars, standard error. (B) Cells from inguinal lymph nodes were cultured for 72 hrs with splenic DC, and T cell proliferation was assessed by incorporation of ^3^H-thymidine. For each mouse, T cells were cultured in triplicate with either DC with gastric membrane or with DC alone. The mean cpm of T cells cultured with DC with antigen was divided by the mean cpm of the same T cells cultured with DC alone to calculate the stimulation index. Data pooled from four independent experiments. In (B), each circle represents the data from one mouse. Mann-Whitney *U* test was used; bars, mean and ***, P<0.001.

To determine if there were elevated immune responses to H^+^/K^+^ ATPase in H/Kα^−/−^ mice relative to wildtype mice, mice were immunised twice with purified gastric H^+^/K^+^ ATPase. After the immunisation, autoantibodies specific for H^+^/K^+^ ATPase were detected in the immunised H/Kα^−/−^ mice, but not in wildtype mice (Figure 1A). T cell response to *in vitro* restimulation with H^+^/K^+^ ATPase was also significantly greater in H/Kα^−/−^ mice than in wildtype mice (Figure 1B). Therefore, as has been previously demonstrated in H/Kβ^−/−^ mice [23], there is also a significant T and B cell response to H^+^/K^+^ ATPase in H/Kα^−/−^ mice that is not present in wildtype mice.

### CD4^+^ T cells from H/Kα^−/−^ mice were highly pathogenic

To determine if adoptive transfer of T cells from H/Kα^−/−^ mice would be able to cause autoimmune gastritis, we enriched CD4^+^ T cells from either H/Kα^−/−^ or wildtype mice to ∼85% purity and transferred 5×10^7^ of these cells into sublethally irradiated wildtype mice. Since it was previously demonstrated that H^+^/K^+^ ATPase-specific CD4^+^ T cells were rapidly deleted in the periphery and unable to induce autoimmune gastritis in non-irradiated wildtype mice [24], all recipient mice used in this study were lightly irradiated at 600 rads to enhance the survival of transferred T cells from H/Kα^−/−^ mice.

**Figure 2:**
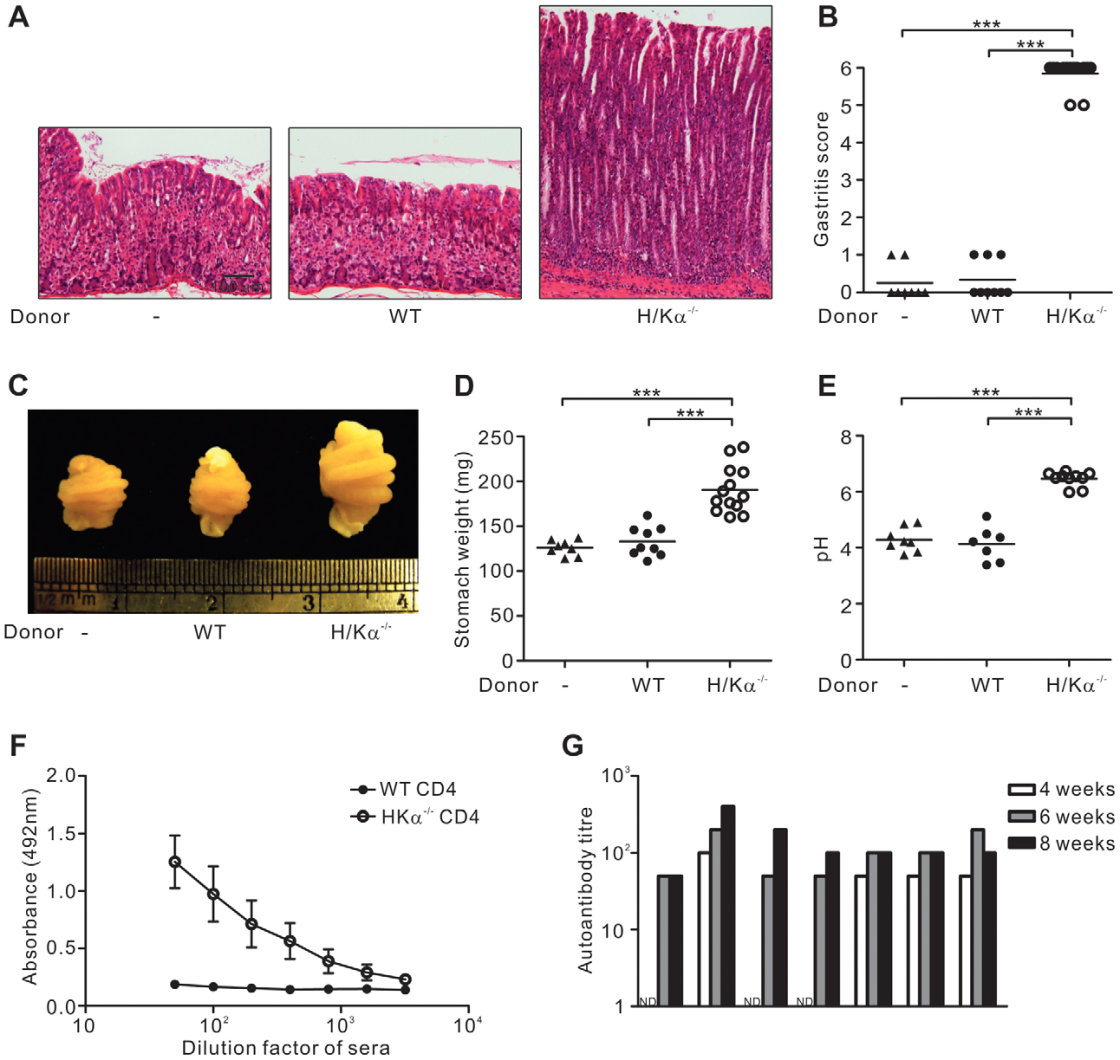
Mice that receive CD4^+^ T cells from H/Kα^−/−^ mice develop severe autoimmune gastritis. Irradiated wildtype mice that received 5×10^7^ CD4^+^ T cells from either wildtype (WT) or H/Kα^−/−^ mice, were killed 8 weeks after transfer and compared to non-manipulated mice (−). (A) Haematoxylin and eosin stained sections of stomachs (B) Gastritis scores. (C) Macroscopic views of stomachs (D) Stomach weights after stomach contents were removed (E) Gastric pH. Mice were starved overnight and their stomachs were rinsed in 1 ml saline which was then collected and measured by a pH meter. (F) H^+^/K^+^ ATPase-specific autoantibodies in serum were detected by ELISA using serial 2-fold dilutions of mouse sera, with a starting dilution factor of 50. Error bars, standard error. (G) Gastric H^+^/K^+^ ATPase autoantibody levels in mice at various times after cell transfer. Serum samples were collected at the indicated time points from each mouse after transfer of CD4^+^ T cells from H/Kα^−/−^ mice. Autoantibody titre was represented by the maximum dilution factor that had an absorbance reading above 50% maximum. ND = not detectable. Data pooled from four independent experiments. In (B), (D) and (E), each circle represents the data from one mouse. Mann-Whitney *U* test was used; bars, mean and ***, P<0.001.

Eight weeks after the transfer, mice that received H/Kα^−/−^ CD4^+^ T cells all developed the most severe forms of autoimmune gastritis (score 5 and 6). This was demonstrated by the complete depletion of parietal cells and zymogenic cells, and hypertrophy of the gastric mucosa (Figure 2A, 2B). In contrast, mice that received wildtype CD4^+^ T cells were completely free of gastritis (Figure 2A, 2B), which indicated that autoimmune gastritis in mice receiving H/Kα^−/−^ CD4^+^ T cells was not caused by irradiation *per se*, but rather, the T cell inoculum.

Pathological changes in the stomach of gastritic mice significantly affected their stomach physiology. Hypertrophy of the gastric mucosa resulted in the enlargement of stomach rugae (Figure 2C) and an increase in stomach weight (Figure 2D). Secondly, depletion of parietal cells caused an increase in stomach pH due to the lack of acid secretion (Figure 2E). In addition, autoantibodies specific to the gastric H^+^/K^+^ ATPase could be detected in all gastritic mice (Figure 2F). Autoantibodies reached high levels in all mice 6 weeks after the transfer (Figure 2G). Together, these results demonstrated the presence of highly pathogenic H^+^/K^+^ ATPase-specific T cells in H/Kα^−/−^ mice.

### Autoimmune gastritis was caused by CD4^+^ T cell-mediated inflammation

**Figure 3:**
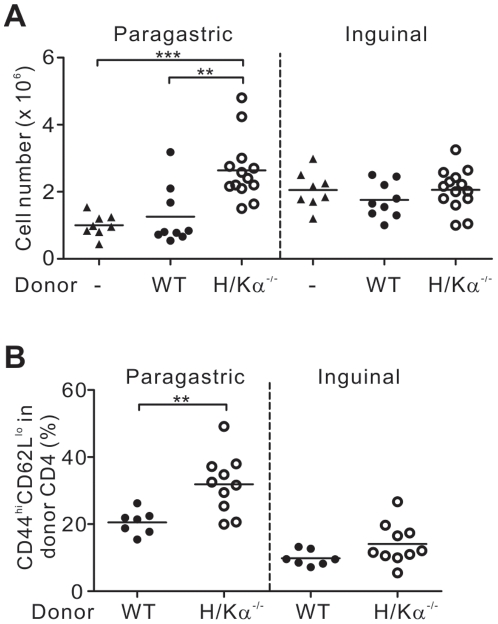
Cellularity of stomach-draining and non-draining lymph nodes. (A) The number of cells in the paragastric and inguinal lymph nodes from recipient mice 8 weeks after transfer of either wildtype or H/Kα^−/−^ CD4^+^ T cells. (B) The percentage of effector/memory T cells in the donor population with the phenotype CD44^hi^CD62L^lo^ in CD90.2^+^ CD4^+^ T cells. Data pooled from four independent experiments. Each circle represents the data from one mouse. Mann-Whitney *U* test was used; bars, mean, **, P<0.01 and ***, P<0.001.

Mice with autoimmune gastritis induced by transfer of H/Kα^−/−^ CD4^+^ T cells had significantly more cells in their stomach-draining paragastric lymph nodes compared to normal mice and mice that received CD4^+^ T cells from wildtype donors. In contrast, there was no difference in the size of the inguinal lymph node between gastritic and non-gastritic mice (Figure 3A). This suggested that the increase in the cellularity of paragastric lymph nodes in gastritic mice was caused by local gastric inflammation and not by preferential survival or expansion of H/Kα^−/−^ CD4^+^ T cells compared to wildtype CD4^+^ T cells after transfer.

In these experiments, cells from the donor H/Kα^−/−^ or wildtype mice could be differentiated from recipient cells because they bore different CD90 alleles. The donor population in the paragastric lymph node of mice that received H/Kα^−/−^ CD4^+^ T cells had significantly more CD4^+^ T cells with an effector/memory phenotype (CD44^hi^CD62^lo^) in comparison to mice that received wildtype CD4^+^ T cells. However, the levels of effector/memory T cells were similar in the non-draining lymph node (Figure 3B). Together, these results suggest that the development of autoimmune gastritis in mice that received CD4^+^ T cells from H/Kα^−/−^ mice was caused by a T cell-mediated inflammatory response in the gastric environment rather than a more generalised response.

**Figure 4:**
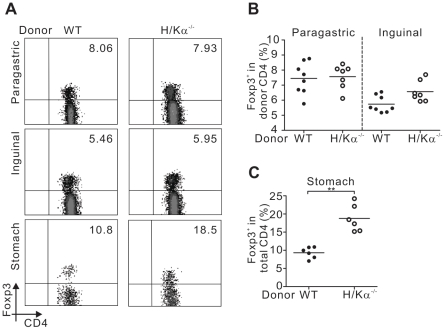
Mice with autoimmune gastritis have an increased level of Foxp3^+^ Treg cells in the stomach. Cells were harvested from (A, B) paragastric lymph node, inguinal lymph node and (A, C) stomach tissue for intracellular staining of Foxp3. The numbers indicate the percentages of Foxp3^+^ Treg cells in donor CD90.2^+^CD4^+^ T cells in paragastric and inguinal lymph nodes and Foxp3^+^ Treg cells in total CD4^+^ T cells in stomach tissue. Data pooled from three independent experiments. In (B) and (C), each circle represents the data from one mouse. Mann-Whitney *U* test was used; bars, mean, and **, P<0.01.

The development of autoimmune gastritis in this disease model was not associated with a reduced level of regulatory T (Treg) cells since gastritic mice contained similar proportions of Treg cells in the paragastric lymph node compared to non-gastritic controls (Figure 4A, 4B). In fact, significantly more Treg cells were found in the stomach of mice with autoimmune gastritis (Figure 4A, 4C).

### H/Kα^−/−^ CD4^+^ T cells rapidly induced tissue damage in the stomach

**Figure 5:**
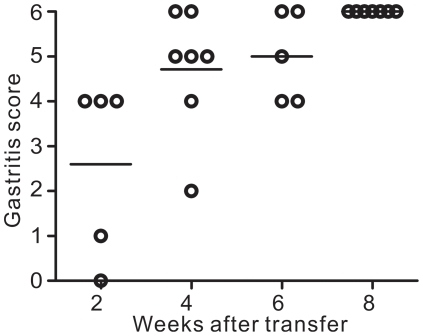
Severity of autoimmune gastritis correlates to stomach weight. Mice were killed at indicated time points after transfer of CD4^+^ T cells from H/Kα^−/−^ mice and the severity of autoimmune gastritis was determined. Data were pooled from six independent experiments. Each circle represents the data from one mouse. Bars, mean.

The severity of autoimmune gastritis was analysed at various time points after the transfer of H/Kα^−/−^ CD4^+^ T cells. Moderate depletion of parietal cells and zymogenic cells, as indicated by a gastritis score of 4 [24], could be observed as early as 2 weeks after the transfer and the disease progressed rapidly with most of the mice showing severe gastritis, as shown by a gastritis score of 5 and 6, 4 weeks after the transfer (Figure 5).

### Stomach weight can be used as a quantitative measure of autoimmune gastritis

**Figure 6:**
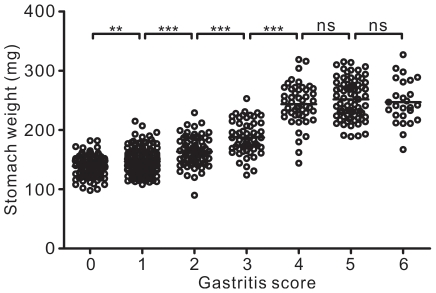
Significant difference in stomach weight among gastritis scores. Thymectomy was performed on BALB.B6-*Gasa* congenic mice on day 3 of age and mice killed at 12 weeks. The severity of autoimmune gastritis and stomach weight was determined. Data pooled from at least 100 independent thymectomies. Each circle represents the data from one mouse. Mann-Whitney *U* test was used; bars, mean, **, P<0.01 and ***, P<0.001.

We have demonstrated that mice with autoimmune gastritis had a greater stomach weight than disease-free mice due to the hypertrophy of the gastric mucosa (Figure 2D). Therefore, we investigated if stomach weight could be used as a quantitative measure for the severity of autoimmune gastritis. To further test if stomach weight could differentiate among different severities of autoimmune gastritis, we examined neonatally-thymectomised mice as greater numbers of mice with variable disease scores could be obtained. We found that there were significant differences in the mean stomach weight between each of the first five levels of autoimmune gastritis (score 0–4) (Figure 6). However, the mean stomach weights of gastritis scores 4, 5 and 6 were not significantly different from each other (Figure 6). This is likely due to the fact that the degree of hypertrophy of gastric mucosa does not increase as the disease progresses to the late stages.

### Induction of autoimmune gastritis by H/Kα^−/−^ CD4^+^ T cells was dependent on the presence of H/Kα and H/Kβ

We hypothesised that the pathogenicity of CD4^+^ T cells from H/Kα^−/−^ mice was caused by the lack of tolerance to H/Kα in the absence of this gastric autoantigen. To test this, CD4^+^ T cells were transferred from H/Kα^−/−^ mice into sublethally-irradiated H/Kα^−/−^ and wildtype mice. It has been demonstrated that H/Kα, in the absence of H/Kβ, cannot be presented by MHC molecules [25] (S Allen, personal communication). We therefore transferred CD4^+^ T cells from H/Kα^−/−^ mice into sublethally-irradiated H/Kβ^−/−^ mice and compared them with H/Kα^−/−^ and wildtype recipient mice. The analysis of autoimmune attack on the gastric mucosa is complicated in H/Kα^−/−^ and H/Kβ^−/−^ mice by the changes that occur in the gastric mucosa since a lack of gastric acid in these mice results in elevated levels of the trophic hormone gastrin. This leads to constitutive hypertrophy and depletion of zymogenic cells [26], . Therefore, in order to assess if an autoimmune response attacks the gastric mucosa in these mice, we instead determined the prevalence of parietal cells, which are normally depleted in autoimmune gastritis but are found in abundance in H/Kα^−/−^ and H/Kβ^−/−^ mice, and also the presence of gastric autoantibodies.

**Figure 7:**
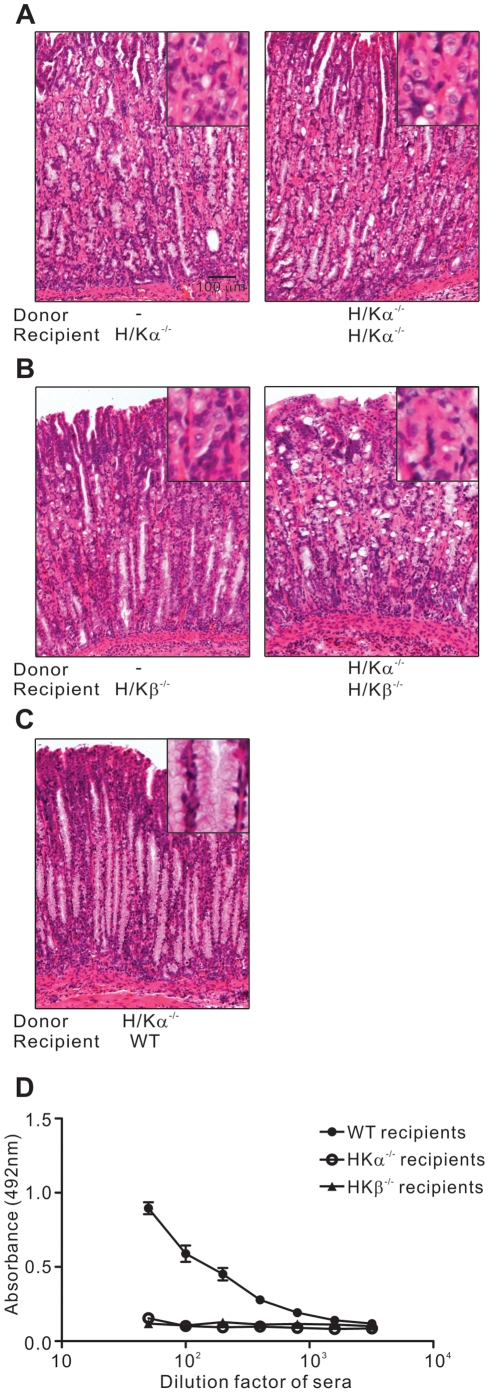
Autoimmune gastritis does not develop in the absence of gastric H/Kα or H/Kβ. Gastric pathology in (A) H/Kα^−/−^ mice received either H/Kα^−/−^ CD4^+^ T cells or no inoculum (−) (B) H/Kβ^−/−^ mice received either H/Kα^−/−^ CD4^+^ T cells or no inoculum (−) (C) WT mice received H/Kα^−/−^ CD4^+^ T cells. Images representative of results of at least 9 mice in each group are shown. (D) Serum samples were collected from H/Kα^−/−^, H/Kβ^−/−^ or WT mice that received CD4^+^ T cells from H/Kα^−/−^ mice. H^+^/K^+^ ATPase-specific autoantibodies in serum were detected by ELISA using serial 2-fold dilutions of mouse sera, with a starting dilution factor of 50. Data pooled from four independent experiments. Error bars, standard error.

The stomach morphology in H/Kα^−/−^ mice or H/Kβ^−/−^ mice was not affected by the transfer of H/Kα^−/−^ CD4^+^ T cells (Figure 7A, 7B). In particular, there was no depletion of parietal cells indicating the lack of an autoimmune response. In contrast, complete depletion of parietal cells was seen in wildtype recipient mice that received H/Kα^−/−^ CD4^+^ T cells (Figure 7C). Since stomachs of H/Kα^−/−^ and H/Kβ^−/−^ mice appeared different to both normal and gastritic mice, we could not assess these mice using our standard gastritis scoring system. However, H^+^/K^+^ ATPase-specific autoantibodies, another hallmark of autoimmune gastritis, were not detected in the sera of both H/Kα^−/−^ and H/Kβ^−/−^ recipient mice, but were detected in wildtype recipient mice (Figure 7D). These results demonstrated that the presence of both α and β subunits was required for the depletion of parietal cells by H/Kα^−/−^ CD4^+^ T cells.

## Discussion

Autoimmune gastritis can be induced in BALB/c mice by several means, including neonatal thymectomy, adult thymectomy with cyclophosphamide [19], [21], immunisation with gastric H^+^/K^+^ ATPase [5] and generation of H^+^/K^+^ ATPase-specific TCR transgenic mice [7], [8]. However, an immune system dominated by T cells with a single specificity as observed in TCR transgenic mice does not resemble normal physiological conditions. Furthermore, thymectomy and immunisation do not confer consistent disease severity and they are technically demanding, which makes it inconvenient to use these models for the analysis of immunotherapies. We therefore developed a new disease model that relied on the transfer of T cells from H/Kα^−/−^ mice, a polyclonal population that contained highly gastritogenic T cells.

We found that immunisation of H/Kα^−/−^ mice with purified H^+^/K^+^ ATPase induced a vigorous T cell response as the result of the lack of T cell tolerance to the gastric autoantigen, which indicated the presence of H^+^/K^+^ ATPase-specific T cells in H/Kα^−/−^ mice. We further demonstrated that transfer of CD4^+^ T cell populations from H/Kα^−/−^ mice induced damage to the stomach tissues of recipient mice soon after transfer. Autoimmune gastritis induced by the transfer of H/Kα^−/−^ CD4^+^ T cells was consistently severe: all the recipient mice developed the most severe disease eight weeks after transfer and their stomach physiology was significantly affected as indicated by the increase in stomach weight and pH. This model is advantageous because the severity and incidence of disease approaches that observed in TCR transgenic mice yet is caused by a polyclonal repertoire, it is technically simple to establish, it does not rely upon perturbation of the normal Treg cell repertoire.

The donor cell population from the H/Kα^−/−^ mice was enriched to ∼85% CD4^+^ T cells by a “negative selection” procedure because the major aim of this work was to produce a simple, inexpensive and convenient disease model. We conclude that it is the CD4^+^ T cells in the inoculum that caused autoimmune gastritis since it has been firmly established by many studies that CD4^+^ T cells and not other cell types are able to initiate autoimmune gastritis [7]–[17]. It is clear that T and B cell populations reactive to the H^+^/K^+^ ATPase are present in the repertoire of normal mice [5], [14]–[16], [21], [24], [28]–[33]. Therefore, we suggest that the H^+^/K^+^ ATPase-specific T and B cells from the recipient are then recruited to the lesion and the recipient-derived B cells are responsible for the production of autoantibodies.

It has been previously documented that CD4^+^ T cells from H/Kβ^−/−^ mice induce autoimmune gastritis in athymic BALB/c mice but not in irradiated wildtype mice [24]. CD4^+^ T cells from H/Kα^−/−^ mice caused gastritis in irradiated wildtype mice suggesting that this population is more pathogenic than CD4^+^ T cells from H/Kβ^−/−^ mice. These data support a more dominant role for immune response against H/Kα in the pathogenesis of autoimmune gastritis, consistent with findings in TCR transgenic mice [7], [8].

Unlike other disease models that utilised Treg cell-depleted polyclonal T cells from wildtype mice to induce autoimmunity [15], it was not necessary to remove Treg cells from H/Kα^−/−^ CD4^+^ T cell population for the induction of severe autoimmune gastritis. Furthermore, the development of autoimmune gastritis in the recipient mice was not caused by a reduced level of Treg cells from the donor population since gastritic mice contained similar proportion of Treg cells in the paragastric lymph node compared to non-gastritic controls. We have previously demonstrated that Treg cells from H/Kα^−/−^ mice were as effective as Tregs cells from wildtype mice in suppressing H/Kα-specific autoreactive T cells [34]. Therefore, our findings here support previous work indicating that T cell repertoires that had not undergone peripheral T cell deletion are unable to be suppressed by the normal Treg cell population [24], [35].

Although a normal level of Treg cells was detected in the paragastric lymph node of gastritic mice, and a significantly higher percentage of Treg cells was found in the stomach of gastritic mice compared to normal mice, Treg cells were not able to prevent or suppress disease. This is consistent with the finding that although Treg cells accumulated in the CNS when experimental allergic encephalomyelitis was triggered [36], they were not able to prevent the expansion and function of myelin oligodendrocyte glycoprotein-specific T cells, since the local inflammatory cytokine milieu rendered pathogenic T cells resistant to Treg suppression.

Depletion of parietal cells is a hallmark of autoimmune gastritis and the main reason that pernicious anemia is a life-threatening disease because parietal cells produce intrinsic factor, the lack of which is fatal. We found that depletion of parietal cells in this disease model was dependent upon the presence of both H^+^/K^+^ ATPase subunits as CD4^+^ T cells from H/Kα^−/−^ mice only induced parietal cell depletion in wildtype mice but not in H/Kα^−/−^ and H/Kβ^−/−^ mice. Parietal cell depletion was not caused by T cell response towards H/Kβ since H/Kα^−/−^ CD4^+^ T cells did not induce parietal cell depletion in H/Kα^−/−^ recipient mice whilst H/Kβ was expressed in these mice [26]. On the other hand, the absence of parietal cell depletion in H/Kβ^−/−^ recipient mice that received H/Kα^−/−^ CD4^+^ T cells reinforced our previous studies suggesting the need for H/Kβ in order for H/Kα to be presented by MHC molecules [22]. Together, these results suggested that parietal cell depletion in this transfer model developed as a consequence of immune responses to H/Kα by CD4^+^ T cells from H/Kα^−/−^ mice. We note that in previous work we have found that parietal cells are not depleted in either the H/Kα^−/−^ nor H/Kβ^−/−^ mice after neonatal thymectomy, which highlights the consistency of this new model with results from this previous, well-established model.

We also demonstrated here that stomach weight can be used as a quantitative measure of the disease severity since hypertrophy of the gastric mucosa was observed in mice with autoimmune gastritis and resulted in the increase in stomach weight [37]. There was a significant correlation between stomach weight and the severity of autoimmune gastritis in mice that received H/Kα^−/−^ CD4^+^ T cells. This correlation was further confirmed in neonatally-thymectomised mice, with the mean stomach weight in each gastritis score being significantly different to another. However, due to the variation within the groups, large sample size will be required if stomach weight is to be used to estimate disease severity. Therefore, histological examination combined with stomach weight can be viewed as complementary measures for assessing the severity of autoimmune gastritis.

We showed that autoimmune gastritis induced by adoptive transfer of H/Kα^−/−^ CD4^+^ T cells represented an excellent disease model for the dissection of autoimmune response and the design of interventionary therapies for autoimmune diseases. In this disease model, autoimmune gastritis was caused by an unfractionated polyclonal T cell population that contained pathogenic T cells specific for a single antigen, and consistent high-grade disease severity is observed in all recipient mice. Moreover, we showed that disease severity could be easily determined by stomach weight, stomach pH and histological examination of stomach section.

## Materials and Methods

### Ethics statement

This work seeks to obtain a more detailed knowledge of autoimmune disease and the immunological mechanisms that prevent autoimmunity in normal individuals. As the immune system is a highly complex, integrated network, animals are required to properly evaluate these issues.

The majority of procedures were clinical tests performed on blood and tissue samples taken from mice that are maintained in excellent housing conditions. Mice were monitored closely and killed if severely affected or distressed. At the termination of each experiment, mice were killed by CO2 asphyxiation or cervical dislocation, which are associated with a minimum of distress.

All experiments are carefully planned to reduce animal numbers and are performed in strict adherence to guidelines dictated by the University of Melbourne's Animal Experimentation Ethics Committee; the Prevention of Cruelty to Animals Act 1986; and the NHMRC/CSIRO/AAC Australian Code of Practice for the Care and Use of Animals for Scientific Purposes (1997).

This work was approved by the University of Melbourne Animal Ethics Committee under project number 0706327.3.

### Mice

BALB/cCrSlc [38], BALB/cCrSlc.CD90.1 congenic, H/Kα^−/−^ [26], H/Kβ^−/−^ [27] and BALB.B6-*Gasa* congenic mice [20] have been previously described. Both H/Kα^−/−^ and H/Kβ^−/−^ mice had been backcrossed greater than 10 times to BALB/cCrSlc. H/Kα^−/−^ mice were crossed to BALB/c.CD90.1 mice to generate H/Kα^−/−^.CD90.1 mice. All mice were maintained in a conventional animal facility at the Bio21 Molecular Science and Biotechnology Institute, The University of Melbourne. All mice and experiments were approved by the University of Melbourne Animal Experimentation Ethics Committee.

### Antibodies and flow cytometric analysis

Anti-CD3-FITC (145-2C11), anti-CD90.1-FITC (HIS51), anti-CD90.2-FITC (30H-12), anti-CD62L-PE (MEL-14), anti-CD4 PerCP (RM4-5), and anti-CD8-APC (53-6.7) were purchased from BD Pharmingen; anti-CD44-APC (IM-7) was purchased from eBioscience. Intracellular staining of Foxp3 was performed using anti-Foxp3-APC (FJK-16S) and Foxp3 Staining Kit (eBioscience) according to the manufacturer's instruction. Flow cytometry was performed on a Becton Dickinson FACSort flow cytometer and analysed using CellQuest Pro software or Beckman Coulter CyAn and analysed using Summit software.

### Assay of T cell responses in mice immunised with H^+^/K^+^ ATPase

Purified pig gastric H^+^/K^+^ ATPase was prepared in a 1∶1 ratio with Freund's complete adjuvant (Life Technologies). Mice were injected subcutaneously at each side of the tail base. H/Kα^−/−^ and BALB/cCrSlc mice were immunised twice with 30 µg of H^+^/K^+^ ATPase, with one month apart between the immunisations. One week after the second immunisation, mice were sacrificed for their sera and inguinal lymph nodes. H^+^/K^+^ ATPase-specific autoantibodies were detected by ELISA. Cells from the inguinal lymph nodes were used in a T cell proliferation assay. 1×10^5^ cells were cultured for 72 hrs with 2×10^4^ splenic DC from BALB/cCrSlc mice. T cell proliferation was assessed by incorporation of ^3^H-thymidine over the final 16 hours of culture. T cells were cultured in triplicate with either DC with antigen (50 µg/ml mouse gastric membrane) or with DC alone. Gastric membranes were prepared from mouse stomachs as previously described [5].

### Induction of autoimmune gastritis in mice

CD4^+^ T cells were enriched to >85% purity from lymph nodes and spleens of H/Kα^−/−^ and BALB/cCrSlc mice. Single cell suspensions were incubated in a mix of F4/80 and B220 hybridoma supernatants plus anti-CD8 antibody (53.6.72, BioXCell). The non-CD4 cells were removed using anti-rat IgG-coated magnetic beads (Dynal, Invitrogen). CD4^+^ (5×10^7^) T cells from either H/Kα^−/−^ or BALB/cCrSlc mice were injected into sublethally irradiated (600 rads) wildtype congenic BALB/cCrSlc.CD90.1 mice via the intraperitoneal route. In some experiments, purified H/Kα^−/−^ CD4^+^ T cells were injected into irradiated BALB/cCrSlc, H/Kα^−/−^ and H/Kβ^−/−^ mice. All recipient mice were irradiated at 600 rads one day before T cell transfer. Eight weeks later, recipient mice were killed and stomachs, lymph nodes and sera were harvested.

BALB/cCrSlc and BALB.B6-*Gasa* congenic mice were used in the thymectomy experiments. Neonatal thymectomy was performed as previously described [20].

### Histological examination of stomachs and measurement of gastric pH

Stomach sections were examined for the presence of pathological changes as previously described [24]. Scoring was performed in a blind fashion and each slide was independently scored by at least two people. In order to measure the stomach pH, mice were starved overnight before sacrifice. Stomachs were cut open and rinsed in 1 ml saline which was collected and measured by a pH meter.

### H^+^/K^+^ ATPase-specific enzyme linked immunosorbent assay (ELISA)

An ELISA to detect H^+^/K^+^ ATPase-specific autoantibodies was carried out using purified pig H^+^/K^+^ ATPase as previously described [29].

### Statistical analysis

Statistical analysis was performed using GraphPad Prism 5.0 (GraphPad). Data were analysed using the Mann-Whitney U-test or Spearman correlation test and a P value<0.05 was considered significant.
